# Stuart has got the PoWeR! Skeletal muscle adaptations to a novel heavy progressive weighted wheel running exercise model in C57BL/6 mice

**DOI:** 10.1113/EP091494

**Published:** 2023-11-16

**Authors:** Pieter J. Koopmans, Therin D. Williams‐Frey, Kevin A. Zwetsloot

**Affiliations:** ^1^ Integrative Muscle Physiology Laboratory Appalachian State University Boone North Carolina USA; ^2^ Department of Public Health and Exercise Science Appalachian State University Boone North Carolina USA; ^3^ Cell and Molecular Biology Program University of Arkansas Fayetteville Arkansas USA; ^4^ Department of Biology Appalachian State University Boone North Carolina USA

**Keywords:** murine exercise models, muscle hypertrophy, resistance exercise

## Abstract

Murine exercise models are developed to study the molecular and cellular mechanisms regulating muscle mass. A progressive weighted wheel running model, named ‘PoWeR’, was previously developed to serve as a more translatable alternative to involuntary resistance‐type exercise models in rodents, such as synergist ablation. However, mice still run great distances despite the added resistance as evidenced by a large glycolytic‐to‐oxidative shift in muscle fibre type. Thus, PoWeR reflects a blended resistance/endurance model. In an attempt to bias PoWeR further towards resistance‐type exercise, we developed a novel heavy PoWeR model (hPoWeR) utilizing higher wheel loads (max of 12.5 g vs 6 g). Adult male C57BL/6 mice voluntarily performed an 8‐week progressive loading protocol (PoWeR or hPoWeR). Running distance peaked at ∼5–6 km day^−1^ in both treatments and was maintained by PoWeR mice, but declined in the hPoWeR mice as load increased beyond 7.5 g. Peak isometric force of the gastrocnemius–soleus–plantaris complex tended to increase in wheel running treatments. Soleus mass increased by 19% and 24% in PoWeR and hPoWeR treatments, respectively, and plantaris fibre cross‐sectional area was greater in hPoWeR, compared to PoWeR. There were fewer glycolytic and more oxidative fibres in the soleus and plantaris muscles in the PoWeR treatment, but not hPoWeR. Collectively, these data suggest hPoWeR may modestly alter skeletal muscle supporting the aim of better reflecting typical resistance training adaptations, in line with decreased running volume and exposure to higher resistance. Regardless, PoWeR remains an effective hypertrophic concurrent training model in mice.

## Abstract (highlights)

- **What is the central question of this study?**
  Can utilizing a heavier loading protocol, compared to the previously developed progressive weighted wheel running (PoWeR) exercise model, bias skeletal muscle adaptations further towards resistance‐type exercise in mice?
- **What is the main finding and its importance?**
  Utilizing a significantly heavier wheel loading protocol, which may better reflect traditional resistance‐type exercise training, only modestly alters skeletal muscle adaptations. These results intuitively demonstrate decreased running distance with exposure to higher resistance, compared to PoWeR; however, murine wheel running models may always inherently possess a strong endurance training component.

## Graphical abstract

We developed a novel heavy PoWeR model (hPoWeR) utilizing higher wheel loads (max of 12.5 g vs 6 g). Adult male C57BL/6 mice voluntarily performed an 8‐week progressive loading protocol (PoWeR or hPoWeR). At the end of the protocol, in situ contractile function determined strength of the posterior plantarflexor muscles; then harvested muscles were assessed for muscle mass, muscle fibre cross‐sectional area, fibre type and anabolic signalling. A summary of results for each intervention is provided. In conclusion, these data suggest hPoWeR modestly induces skeletal muscle alterations that better reflect typical resistance training adaptations, in line with decreased running volume and exposure to higher resistance. Figure generated using BioRender.com.

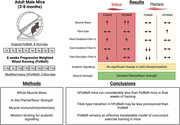

## 1 INTRODUCTION

Skeletal muscle comprises ∼40% of body mass in adult humans and plays an integral role in whole body energy metabolism, glucose homeostasis and movement (Frontera & Ochala, 2015). Unfortunately, humans suffer from a largely inevitable age‐related loss of muscle mass (∼1% per year after the age of 50) leading to a decline in function and vitality (Larsson et al., 2019). Exercise is the most efficacious strategy for maintaining skeletal muscle health throughout the lifespan. Identifying the underlying mechanisms regulating muscle mass and function with exercise adaptation, particularly during ageing, is of critical importance so researchers can develop targeted therapies to promote healthy ageing in humans.

To investigate the mechanisms regulating muscle mass, many resistance‐type exercise models have been developed for use in rodents, including synergist ablation, electrical stimulation, weighted ladder climbing, weighted pulling and canvassed squatting (Cholewa et al., 2014; Murach, McCarthy et al., 2020). While these models are valuable, they are also invasive, tedious, involuntary, and require sufficient manpower. Fortunately, many rodent strains voluntarily run long distances when given access to a running wheel and do not rely on positive/negative reinforcement or anaesthesia to force movement (Goh & Ladiges, 2015; Lightfoot et al., 2004). There is a strong influence of genetic background on running performance, where the commonly used C57 strains will run ∼5–8 km day^−1^, with female mice running ∼20% farther than males, perhaps due to lower body mass (Lightfoot et al., 2004, 2010).

Leveraging the voluntary wheel running behaviour in mice, Dungan et al. (2019) developed a high‐throughput translatable model of progressive weighted wheel running in mice (PoWeR; Dungan et al., 2019, 2022; Murach, McCarthy et al., 2020). Unlike traditional constant‐friction loaded wheel running models, progressive loading is applied asymmetrically to the wheel by adhering mass/load (e.g., weighted magnets) to one side of the wheel, resulting in an unbalanced loading pattern. This causes mice to run in a more intermittent pattern, as opposed to traditional loading wheel running models (Konhilas et al., 2005; Soffe et al., 2016) – more akin to traditional resistance exercise (e.g., sets and reps). PoWeR also has the advantage of training large cohorts of mice with minimal intervention from the researcher. PoWeR includes an acclimation week with zero added load, then load is progressively increased up to 6 g over an 8‐week protocol. In both young and old mice, PoWeR successfully induces whole tissue skeletal muscle growth, muscle fibre hypertrophy, myonuclear accretion, modest strength improvements and enhanced capillarization. Further, unlike models such as synergist ablation, the adaptations produced by PoWeR occur over a time frame similar to human exercise training and are reversible after periods of detraining (Dungan et al., 2019; Murach, Mobley et al., 2020; Wen et al., 2021). However, despite the addition of 6 g of load, mice maintain high running volumes in this model. Young female mice (4‐6 months old) run approximately 10–12 km day^−1^ and old female mice (22–24 months old) run 6–8 km day^−1^, concomitantly promoting a robust glycolytic‐to‐oxidative shift in fibre myosin heavy chain (MyHC) composition (Dungan et al., 2019; Murach, Mobley et al., 2020; Valentino et al., 2021). Therefore, PoWeR must be utilized with the understanding that it reflects a more blended resistance/endurance exercise model (e.g., concurrent training).

The purpose of this investigation was to determine if utilizing considerably greater wheel load than used in the PoWeR model can bias this voluntary concurrent training model more towards resistance‐type exercise training, while inducing greater strength and hypertrophic outcomes, compared to PoWeR. We aimed to: (1) characterize the running patterns (distance and speed) of mice utilizing approximately twice the load used in PoWeR and (2) assess strength, muscle hypertrophy, fibre type (FT) and anabolic signalling outcomes in response to progressive weighted wheel running.

## 2 METHODS

### 2.1 Ethics approval

This study was approved by the Appalachian State University IACUC (no. 22‐05) in compliance with the Animal Welfare Act, the Public Health Service Policy on Humane Care and Use of Laboratory Animals, and our Animal Welfare Assurance. We understand the ethical principles under which the journal operates and our work complies with the journal's animal ethics checklist.

### 2.2 Animals

Thirty‐seven male C57BL/6 mice were procured from our in‐house animal facility and randomly assigned to three treatments: sedentary (SED, *n* = 13), PoWeR (*n* = 13) and heavy PoWeR (hPoWeR, *n* = 11). Mice that did not show an inclination to run within the first week were excluded from analyses, reducing sample sizes to *n* = 10 for both PoWeR and hPoWeR. Mice were at the age of 3–8 months at the start of the study as daily running activity plateaus around 9–10 weeks (Swallow et al., 1998). All mice were individually housed in a cage with a running wheel and kept in a controlled environment with a 12:12‐h light–dark cycle. Standard rodent chow and water were available ad libitum and food consumption was measured daily. Body mass was recorded weekly. The running wheel was locked 24 h prior to contractile function analyses and tissue harvest.

### 2.3 Wheel running apparatus

The cage set‐up and loading protocol are described in detail in a previous publication by our lab (Koopmans & Zwetsloot, 2022). Briefly, all mice had free access to a running wheel 11.4 cm in diameter with an 8 cm‐wide running surface (Kaytee Silent Spinner Exercise Wheel, Petco, Inc., San Diego, CA, USA). Each cage was equipped with a digital bike computer (Sigma Sports, BC 509, Hampton Wick, UK) to monitor time exercising, distance travelled (set at 0.358 m revolution^−1^), and average speed (km h^−1^). The bike computer was observed daily at a consistent time interval to record all data. A small magnet adhered to the middle circumference of the wheel functioned as a sensor for the bike computer to detect each wheel revolution.

The entire protocol was conducted over a 9‐week period. For mice in the PoWeR and hPoWeR treatments, week 1 consisted of an acclimation phase to allow the mice to become accustomed to running on the wheel with no load (e.g., free wheel). Mice in the SED treatment had a locked wheel in their cage to prevent running. Starting on week 2, mice in the PoWeR and hPoWeR treatments began their progressive 8‐week wheel progressive loading protocols, respectively (Table 1).

**TABLE 1:** Loaded wheel running protocols.

	Week								
	1	2	3	4	5	6	7	8	9
PoWeR loading protocol									
Load (g)	0	2	3	4	5	5	6	6	6
% BM	0	8	11	15	19	19	23	23	23
Novel heavy PoWeR loading protocol									
Load (g)	0	2.5	5	7.5	7.5	10	10	12.5	12.5
% BM	0	10	19	28	28	38	38	48	48

*Note*: The PoWeR loading protocol is from Dungan et al. (2019). Load progression in the PoWeR protocol is identical to the original PoWeR model (developed by Dungan et al., 2019); the heavy PoWeR protocol load progression is based on the original PoWeR model. Percentage body mass (% BM) is the result of added load divided by approximate average body mass of adult male C57BL/6 mice.

### 2.4 in situ muscle function

At the end of the 9‐week protocol, an in situ contractile function procedure was performed to determine strength of the posterior plantar flexor muscles of the hindlimb for each mouse according to previously published methods (Mackay et al., 2021). First, the animal was deeply anaesthetized with 4% isoflurane at 1 litre min^−1^ O_2_ flow and anaesthesia was maintained with 2% at 0.5 litre min^−1^ O_2_ flow isoflurane for the duration of the procedure. The mouse was then placed on a heated platform to maintain body temperature at 37°C.

Using only the right limb, the hamstring muscle and excess tissue were dissected away to expose the sciatic nerve and to isolate the gastrocnemius–plantaris–soleus (GPS) muscle complex (aka, the posterior plantar flexor muscles). The distal Achilles tendon and the GPS complex of the right limb were then released up to its proximal origin to mitigate any lateral transmission of force from nearby muscles. The peroneal nerve, which innervates the anterior crural muscles, was also transected to minimize movement of the foot. This procedure leaves all innervation and vasculature to the GPS complex intact. To prevent movement of the knee joint, the patella tendon was secured to a vertical post on the heated platform by 2‐0 surgical suture.

The Achilles tendon, with a part of the calcaneus bone still attached to alleviate slipping, was then secured to a 4 cm lever arm connected to the servomotor of the Aurora Scientific 1305 Muscle Testing System via 2‐0 suture (Aurora Scientific, Toronto, ON, Canada). Two 30 G microelectrode needles were placed superficially staggering the sciatic nerve. Electrode placement was optimized using single electrical pulses (10 mA) to produce muscle contractions of the GPS complex. Resting muscle length was adjusted very carefully until resting tension reached 0.1 N (Weber et al., 2012). Following optimal muscle length and stimulation amplitude, measurement of isometric muscle strength of the GPS complex was performed using a force–frequency curve with 11 ascending stimulation frequencies ranging from 1 Hz to 300 Hz, with 2 min rest between each contraction. The force–frequency curve is designed to assess muscle strength from submaximal, unfused contractions up to maximal, fused tetanic contractions, with maximal fused tetanic contractions typically observed between 100 and 200 Hz. This specific force–frequency curve was published by Corona et al. (2008) and has been successfully conducted in our lab in previous experiments (Corona et al., 2008; Godwin et al., 2020; Zwetsloot et al., 2021).

### 2.5 Tissue harvesting

Immediately following the in situ contractile function procedures, anaesthetized mice were humanely killed by cervical dislocation and death was confirmed via cardiac excision, and then tissues were harvested. For histological purposes, the gastrocnemius, plantaris, soleus and triceps brachii muscles from the left limb were excised and cleared of excess connective tissue and debris, then tissue mass was recorded and individually preserved for histological analyses. Each histological muscle sample was coated in an embedding medium (Tissue‐Tek OCT; Miles, Naperville, IL, USA), mounted on cork, cryopreserved in liquid nitrogen‐cooled isopentane, and stored at −80°C until further use. For cell signalling analyses, the plantaris and soleus muscles from the right limb were flash‐frozen in liquid nitrogen and stored at −80°C until further use.

### 2.6 Immunohistochemical analysis

Muscle tissues were sectioned transversely (10 µm thick) at −16°C using a cryostat (Cryostar NX50, Thermo Fisher Scientific, Waltham, MA, USA) placed on charged slides and then stored at −20°C. Immunohistochemistry against the extracellular matrix protein laminin, myosin heavy chain (MyHC) I, MyHC IIA and MyHC IIB was performed to detect the muscle fibre membrane and muscle fibre‐type, respectively. MyHC IIX was left unstained. Briefly, slides were removed from the freezer and allowed to warm to room temperature. The tissue sections were encircled using a hydrophobic‐barrier PAP Pen (ImmEdge pen, cat. no. H‐4000, Vector Laboratories, Burlingame, CA, USA). Tissue sections were blocked by incubating with approximately 50 µL of 10% normal goat serum (NGS; Vector Laboratories, S‐1000) in 1× phosphate‐buffered saline (PBS) at room temperature for 1 h. Blocking buffer was carefully removed via aspiration. Tissues were incubated overnight at 4°C with a primary antibody cocktail of 1:250 laminin IgG (EMD Millipore, Billerica, MA, USA, 05‐206), 1:200 MHC I IgG2b (Developmental Studies Hybridoma Bank (DSHB), Iowa City, IA, USA, BA‐F8), 1:25 MHC IIA IgG1 (Iowa DSHB, SC‐71), and 1:10 MHC IIB IgM (Iowa DSHB, BF‐F3). After primary antibody incubation, primary antibodies were removed by aspiration and the PAP pen ring was reapplied, if needed. Tissues were washed for three rounds of 5 min with approximately 50 µL of 0.04% Triton X‐100 in 1× PBS (wash buffer). Tissues were then incubated in a secondary antibody cocktail of 1:1500 goat anti‐mouse IgG (Alexa Fluor 405, blue), 1:2000 goat anti‐mouse IgG2b (Alexa Fluor 555, red), 1:150 goat anti‐mouse IgG1 (Alexa Fluor 633, magenta) and 1:100 goat anti‐mouse IgM (Alexa Fluor 488, green) for 1 h. Tissues were again washed for three rounds of 5 min with approximately 50 µL of 0.04% Triton X‐100 in 1× PBS. After fully air drying, coverslips were applied using Vectashield Vibrance anti‐fade mounting medium (Vector Laboratories, H‐1700).

### 2.7 Image analysis

All tissue sections were imaged using a confocal laser scanning microscope (Zeiss LSM 880 with Airyscan; Carl Zeiss Microscopy, Oberkochen, Germany) with Zen software at ×10 magnification. Images of the sections were digitized and MyHC‐fibre type (FT), fibre cross‐sectional area (fCSA), and FT‐specific CSA were determined using the semi‐automated software Myovision (Wen et al., 2018). At least 200 fibres were measured during histological analyses. Whole muscle fCSA, percentage FT, and FT‐specific CSA are reported.

### 2.8 Western blot analysis

The entire soleus and plantaris muscles from the right limb of each mouse were homogenized on ice in 0.3 mL of homogenization buffer containing 1.0 mM NaCl, 1.0% Triton X‐100, 0.5% sodium deoxycholate, 10 mM Tris–HCl, pH 7.6 with protease and phosphatase inhibitor cocktails (Sigma‐Aldrich, St Louis, MO, USA). After homogenization, the samples were cleared by centrifugation for 15 min at 5000 *g*. The supernatant was removed, and protein concentration was determined using the BCA protein assay (Pierce cat. no. 23227; Thermo Fisher Scientific). Total protein (10 µg) was prepared in Laemmli buffer and subjected to electrophoretic separation by SDS‐PAGE on 4%–15% TGX Stain‐free acrylamide gels (Bio‐Rad Laboratories, Hercules, CA, USA). Following electrophoretic separation, gels were exposed to ultraviolet light for crosslinking and then the proteins were transferred to a polyvinylidene difluoride membrane and imaged to ensure equal loading and transfer of proteins. Briefly, non‐specific binding was blocked with 5% non‐fat dry milk in Tris‐buffered saline with 0.1% Tween 20 (TBS‐T) for 1 h at room temperature. Following blocking, membranes were incubated with the following primary antibodies in 5% bovine serum albumin (BSA) in TBS‐T overnight at 4°C: phosphorylated ribosomal protein S6 (p‐rpS6 Ser240/244, 1:1000; cat. no. 5364; Cell Signaling Technology, Danvers, MA, USA) and total rpS6 (1:1000; cat. no. 2217, Cell Signaling Technology). After primary antibody incubation, membranes were washed three times and then incubated for 1 h with horseradish peroxidase (HRP)‐linked IgG anti‐rabbit (1:10,000; Cell Signaling Technology, cat. no. 7074) secondary antibody. Finally, membranes were washed three times and immediately exposed to SuperSignal™ West Dura ECL chemiluminescent detection HRP reagent (Pierce cat. no. 35071; Thermo Fisher Scientific) for 5 min. Membranes were imaged using the ChemiDoc XRS+ molecular imaging system (Bio‐Rad) and the densitometry of specific protein target bands was determined using ImageLab software (Bio‐Rad).

### 2.9 Statistical tests

All data were analysed using SPSS Statistics (Version 28.0.0.0.; IBM Corp., Armonk, NY, USA). One‐way ANOVA was used for food consumption, muscle mass, peak muscle strength, mean fCSA, fibre‐type and anabolic signalling for comparisons between treatments. Body mass and weekly average running distance and speed for each treatment were analysed with repeated measures two‐way ANOVA. Following a significant *F*‐ratio, Fisher's LSD *post hoc* analysis was performed. Significance was set a priori at *P* < 0.05. Effect sizes were estimated using partial eta squared (η^2^) and were interpreted as small effect, η^2^ = 0.010–0.059; medium effect, η^2^ = 0.060–0.139; and large effect, η^2^ > 0.140. Data are expressed as means ± SD. Figures were generated in GraphPad Prism version 9.0 (GraphPad Software, Boston, MA, USA).

## 3 RESULTS

### 3.1 Animal characteristics

**FIGURE 1:**
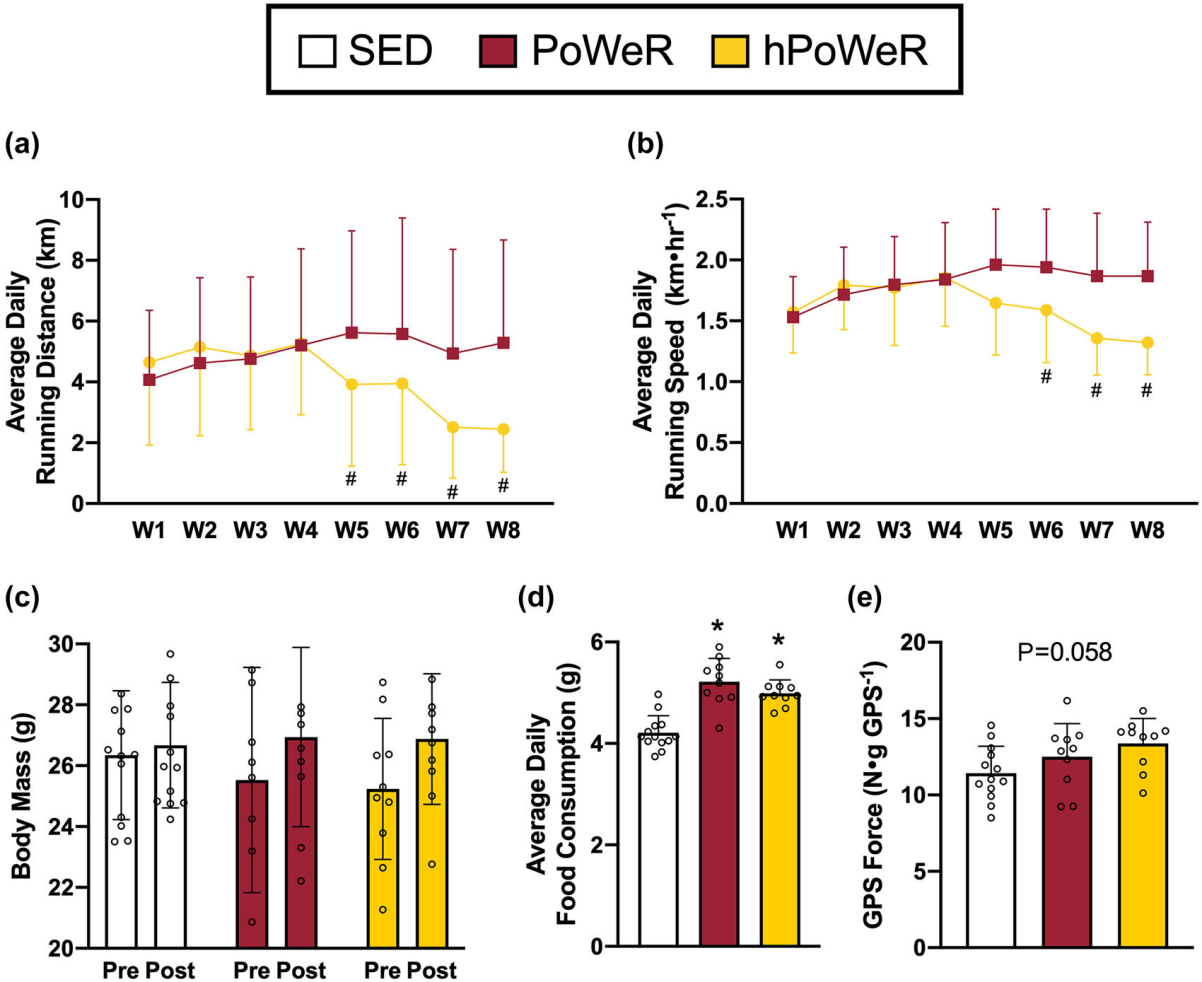
Running distance, running speed, body mass, food consumption and peak GPS force following PoWeR and hPoWeR. Sedentary (SED; white), PoWeR (cardinal red), hPoWeR (gold). (a,b) PoWeR and hPoWeR average daily running distance (a) and average daily running speed (b) after the acclimation week. (c) Body mass of SED, PoWeR, and hPoWeR before and after treatment. (d) Mean daily food consumption of SED, PoWeR and hPoWeR mice. (e) Peak GPS force of SED, PoWeR and hPoWeR mice measured by in situ muscle function test. *Significantly different from SED; #significantly different from PoWeR; *n* ≥ 10 per treatment for each analysis.

Thirty‐three male mice aged 5.8 ± 1.1 months at the start of exercise were included in the analyses: SED (*n* = 13), PoWeR (*n* = 10) and hPoWeR (*n* = 10). Mean body mass of all mice at the start of training was 25.8 ± 2.7 g with no differences between treatments (*P* = 0.605). There was no significant overall effect on body mass (*P* = 0.295), but there was a significant effect of time on body mass (*P* < 0.0001), as body mass significantly increased by ∼1 g over the 9‐week protocol (Figure 1c). Mean daily food consumption significantly increased by ∼20% (*P* < 0.0001) from 4.20 g day^−1^ in SED mice to 5.22 g day^−1^ and 4.99 g day^−1^ in PoWeR and hPoWeR mice, respectively; however, no differences were observed between running wheel treatments (*P* = 0.227, Figure 1d).

### 3.2 Running characteristics

After the acclimation week (week 1), there was a significant overall effect on running distance (*P* < 0.0001). In PoWeR mice, running distance peaks in week 4 and 5 at ∼5–6 km day^−1^ and was maintained for the remainder of the training protocol (Figure 1a). In hPoWeR mice, after week 4 (>7.5 g of load), running distance was lower compared to PoWeR mice. There was also a significant overall effect on running speed (*P* < 0.0001), where hPoWeR running speed was lower compared to PoWeR in weeks 6 and beyond (Figure 1b).

### 3.3 in situ contractile function

Normalized in situ peak force of the GPS complex (N g GPS^−1^) tended to increase +9.5%, +17.0% in PoWeR and hPoWeR, respectively (*P* = 0.058, η^2^ = 0.173, Figure 1e).

### 3.4 Tissue mass

**FIGURE 2:**
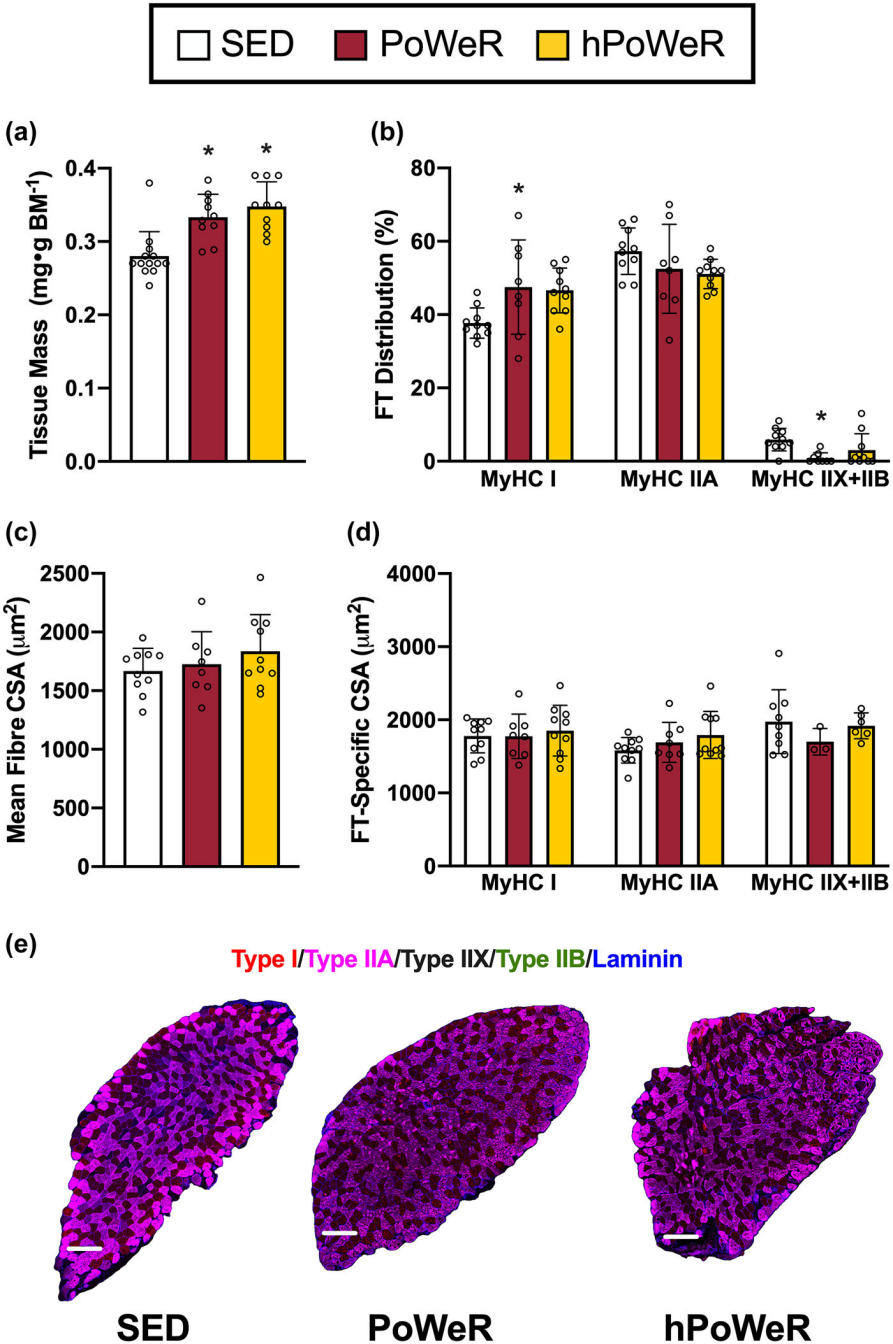
Soleus muscle mass, fibre type (FT) and fibre size following PoWeR and hPoWeR. Sedentary (SED; white), PoWeR (cardinal red), hPoWeR (gold). (a) SED, PoWeR and hPoWeR soleus wet tissue mass normalized to body mass. (b) Soleus fibre type distribution. (c) Soleus mean fibre cross‐sectional area (CSA). (d) Soleus fibre type‐specific CSA (MyHC I, MyHC IIA, MyHC IIX+IIB). (e) Representative images of fibre type and laminin staining of entire soleus muscle cross sections from SED, PoWeR and hPoWeR mice; scale bar, 200 µm; red, type I; magenta, type IIA; unstained/black, type IIX; green, type IIB. *Different from SED; #different from PoWeR; *n* ≥ 8 per treatment for each analysis.

**FIGURE 3:**
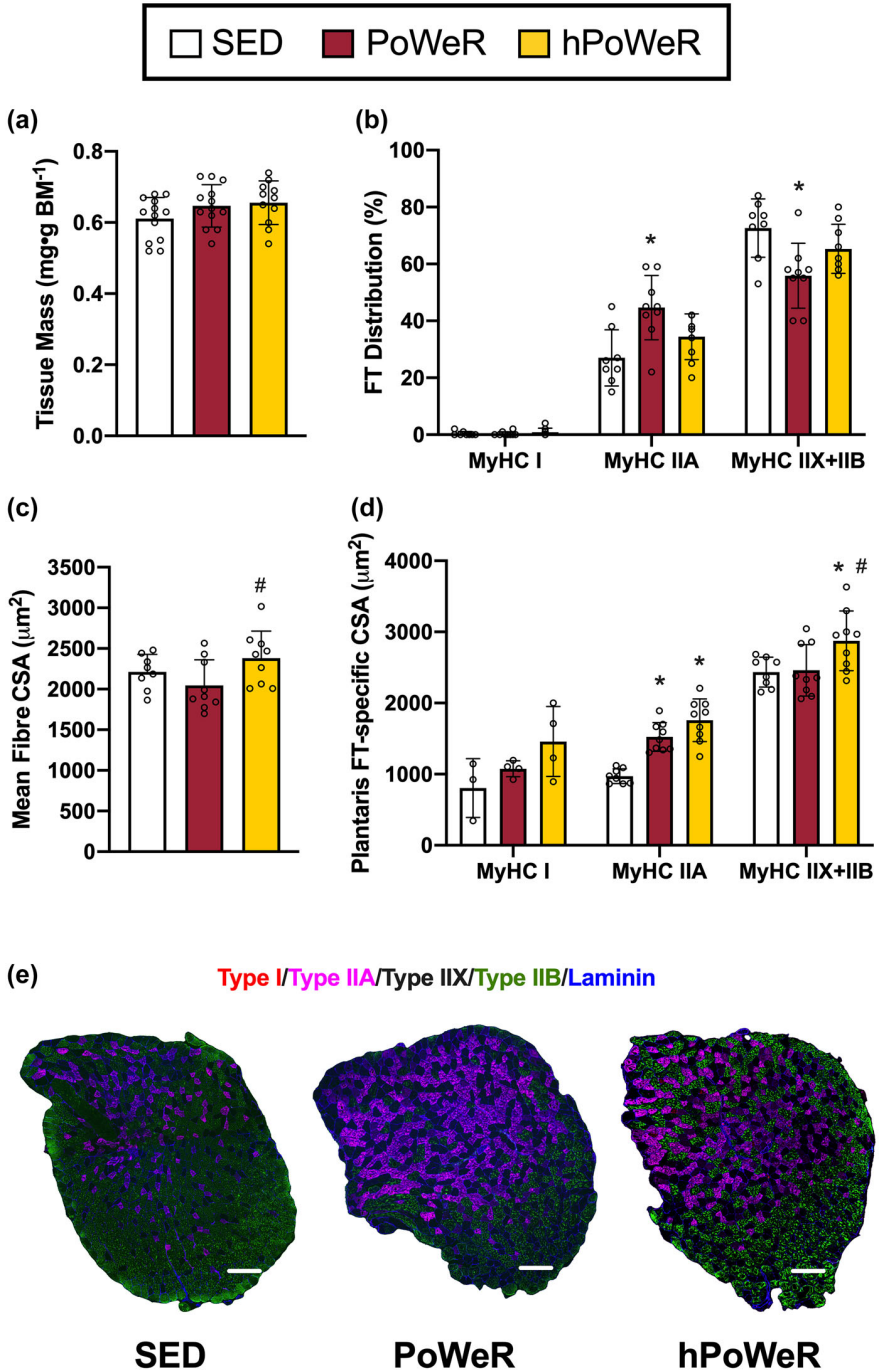
Plantaris muscle mass, fibre type (FT) and fibre size following PoWeR and hPoWeR. Sedentary (SED; white), PoWeR (cardinal red), hPoWeR (gold). (a) SED, PoWeR and hPoWeR plantaris wet tissue mass normalized to body mass. (b) Plantaris fibre type distribution. (c) Plantaris mean fibre cross‐sectional area (CSA). (d) Plantaris fibre type‐specific CSA (MyHC I, MyHC IIA, MyHC IIX+IIB). (e) Representative images of fibre type and laminin staining of entire plantaris muscle cross sections from SED, PoWeR and hPoWeR mice; scale bar, 200 µm; red, type I; magenta, type IIA; unstained/black, type IIX; green, type IIB. *Different from SED; #different from PoWeR; *n* ≥ 8 per treatment for each analysis.

Soleus mass was 18.9% and 23.6% greater in the PoWeR and hPoWeR mice, respectively, compared to SED mice (Figure 2a, both *P* = 0.000578 and *P* < 0.0001, respectively); however, plantaris mass was not significantly different between treatments (Figure 3a *P* = 0.123, η^2^ = 0.301). No significant differences were observed in heart, GAS or triceps brachii (TRI) tissue masses between treatments (data not shown).

### 3.5 Fibre cross‐sectional area

There was a significant increase in whole muscle mean fCSA in the plantaris in hPoWeR compared to the PoWeR (+16.2% or +333 µm^2^, Figure 3c, *P* = 0.00462, η^2^ = 0.303), but no significant differences in the soleus (Figure 2c, *P* = 0.367, η^2^ = 0.077). There were no changes in whole tissue mass for the GAS and TRI; therefore, fCSA was not analysed in these tissues.

### 3.6 Muscle fibre type

In the soleus there was a significant difference in percentage of MyHC I fibres (Figure 2b, *P* = 0.0259, η^2^ = 0.253). *Post hoc* tests indicate PoWeR mice have a greater percentage of MyHC I fibres (+9.8% relative to SED, *P* = 0.0175) and hPoWeR mice also have a greater percentage of MyHC I fibres (+8.9% relative to SED, *P* = 0.0216). There were no significant differences in percentage of MyHC IIA fibres in the soleus (*P* = 0.211, η^2^ = 0.120); however, PoWeR mice have a lower percentage of MyHC IIX+IIB fibres (−5.0% relative to SED, *P* < 0.0001).

In the plantaris (Figure 3b), there were no differences in percentage of MyHC I fibres between treatments (*P* = 0.599). There was a significantly greater percentage of MyHC IIA fibres (+17.5%; *P* = 0.00131) and a significantly lower percentage of MyHC IIX+IIB fibres (−17.6%; *P* = 0.00181) in PoWeR mice compared to SED mice. Fibre type distributions were not significantly different in plantaris of hPoWeR mice.

### 3.7 Fibre type‐specific CSA

CSA was further interrogated at the FT‐specific level. In the plantaris, MyHC IIA fibres in PoWeR and hPoWeR mice were larger than SED (Figure 3d, +551 and +786 µm^2^, respectively; *P* < 0.0001 for both). MyHC IIX+IIB fibres in hPoWeR plantaris were also significantly larger than both SED and PoWeR (+440 and +414 µm^2^, respectively, both *P* < 0.0001). All other FTs in the plantaris and soleus (Figure 2d) displayed no significant differences in CSA. It is worth noting not all tissue sections contained all fibre types, specifically MyHC IIX+IIB fibres in the soleus and MyHC I fibres in the plantaris, so statistical analyses for those specific tissue/fibre type combinations should be interpreted in that light.

### 3.8 Anabolic cellular signalling

**FIGURE 4:**
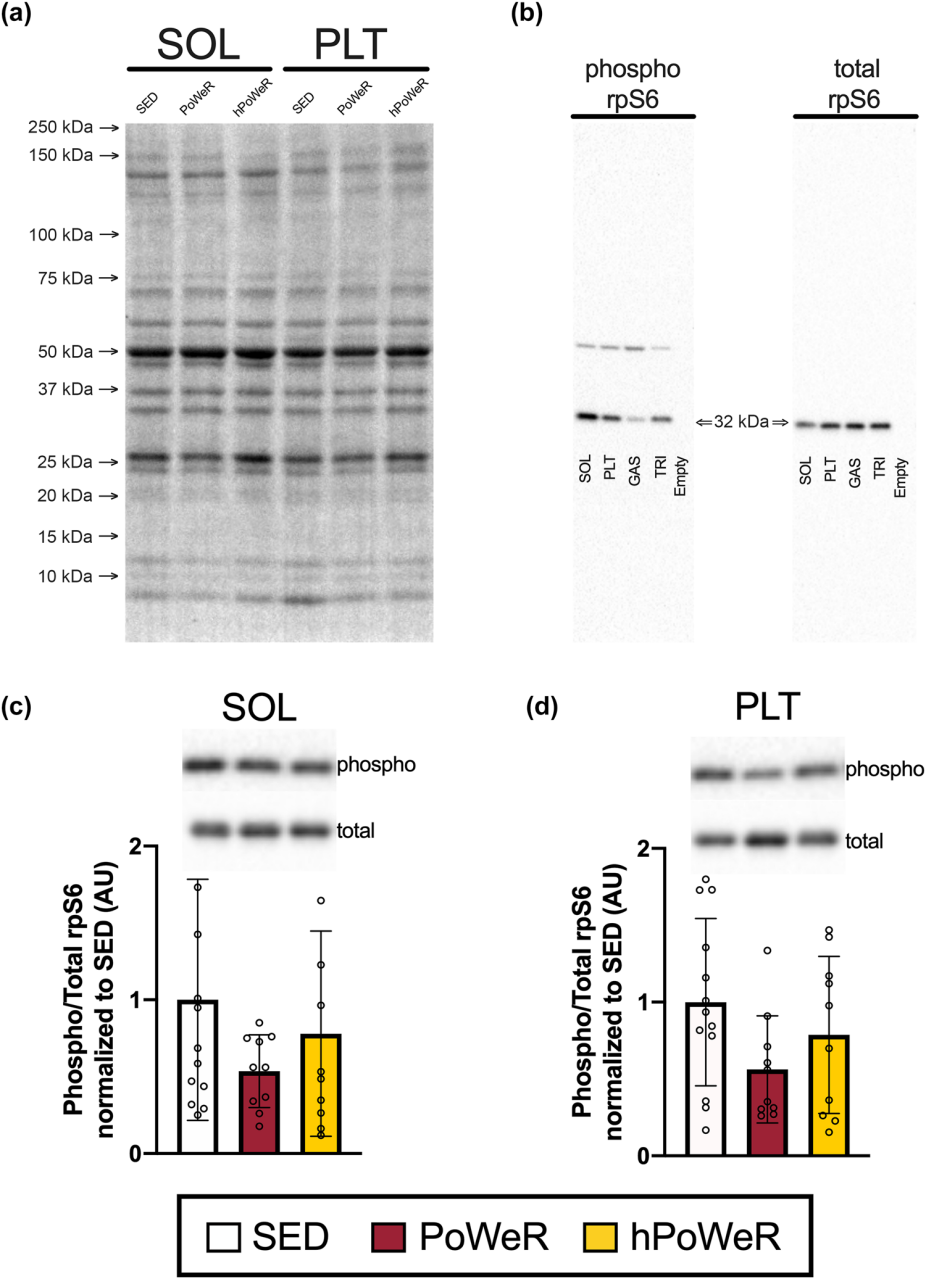
Western blot analysis of the phosphorylation status of ribosomal protein S6 (rpS6) in soleus (SOL) and plantaris (PLT) muscles as measured by ratio of phosphorylated rps6 to total rps6. Sedentary (SED; white), PoWeR (cardinal red), hPoWeR (gold). (a) Representative image of TGX stain‐free blot demonstrating equal protein loading across lanes. (b) Images of preliminary western blots indicating the specificity of the phosphorylated rpS6 antibody (left) and the total rpS6 antibody (right) in different skeletal muscle tissue homogenates, including negative controls (empty well). (c) Phosphorylated‐rps6: total rps6 in soleus muscle of SED, PoWeR and hPoWeR. Upper: representative images of western blots. Lower: quantification of western blot data. (d) Phosphorylated rps6: total rps6 in plantaris muscle of SED, PoWeR and hPoWeR. Upper: representative images of western blots. Lower: quantification of western blot data. *n* ≥ 10 per treatment for all analyses. AU, arbitrary units; GAS, gastrocnemius muscle; PLT, plantaris muscle; SOL, soleus muscle; total, total protein; TRI, triceps muscle; phospho, phosphorylated protein.

As an indicator of long‐term anabolic cellular signalling, the phosphorylation status of ribosomal protein S6 (rpS6; a downstream target of the phosphoinositide 3‐kinase–AKT–mechanistic target of rapamycin (mTOR) axis) was measured. Results from western blot analyses revealed that there were no significant differences in phosphorylation status of rpS6 in either the soleus or plantaris between treatments (*P* = 0.231 and *P* = 0.114, respectively; Figures 4a,b).

## 4 DISCUSSION

PoWeR is a translatable and high‐throughput exercise training model for inducing pronounced skeletal muscle adaptations in young and aged mice. In this investigation, we developed a novel hPoWeR model that modifies the original PoWeR model to use considerably higher wheel loads. We aimed to characterize differences in running patterns, muscular strength, size, fibre type and anabolic signalling outcomes between these models. The major findings of this investigation are: (1) fibre type transitions in our hPoWeR mice were blunted, compared to PoWeR mice, while all other muscular adaptations were quite similar, and (2) running distance is significantly lower in hPoWeR mice than PoWeR mice in the final weeks of training.

When given access to a running wheel, mice tend to develop more consistent running in the first few weeks of training (Allen et al., 2001; Manzanares et al., 2018). Consistent with prior investigations, PoWeR mice reached a peak running distance and speed in week 4, which was maintained for the remainder of the protocol (Figure 1a; Collao et al., 2023; Dungan et al., 2019, 2022; Valentino et al., 2021). Conversely, hPoWeR mice reached a similar peak in running distance and speed, which then abruptly declined as load increased beyond 7.5 g to 12.5 g to approximately half the distance as PoWeR (Figure 1a,b). Lower running speed with the increasing loads also indicates an even more intermittent running pattern than typically seen in PoWeR. Thus, wheel loads up to 7.5 g are likely insufficient to deter mice from running great distances with the asymmetrical loading strategy. We further support this conclusion by finding PoWeR mice run similar distances as mice performing unloaded wheel running (4–6 km day^−1^; data not shown).

Sufficient volumes of exercise training elicit some degree of fibre‐type transitions, the direction of which appears to be dependent on training modality (Plotkin et al., 2021; Wilson et al., 2012). In agreement with previous PoWeR studies, we report the soleus and plantaris of PoWeR‐trained mice display a fast‐to‐slow FT transition, namely a higher percentage of MyHC I fibres and near elimination of MyHC IIX+IIB fibres in the soleus and higher percentage of MyHC IIA and lower percentage of MyHC IIX+IIB fibres in the plantaris (Dungan et al., 2019, 2022; Hammarström et al., 2020; Murach et al., 2020). Remarkably, though, the soleus and plantaris muscle of hPoWeR mice did not display major differences in MyHC fibre type, compared to SED mice. We attribute this blunted fibre type transition in hPoWeR mice to the vastly lower running distances than PoWeR mice. This dovetails with the observation of a dose‐dependent relationship between training volume and magnitude of fibre type transitions in multiple exercise training modalities and species (Demirel et al., 1999).

PoWeR also elicits muscle type‐specific adaptations, where the slow‐oxidative soleus typically displays more robust hypertrophic adaptations than the fast‐glycolytic plantaris in young and old mice (Dungan et al., 2019, 2022; Murach, Mobley et al., 2020). Soleus muscle growth in PoWeR and hPoWeR was similar in magnitude to prior reports, but plantaris mass was unchanged and fibre hypertrophy was limited to MyHC IIA and IIX+IIB fibres in the plantaris. Attenuated whole muscle growth of the plantaris and muscle fibres could be attributable to lower running volumes overall than prior investigations (5–6 km day^−1^ vs 10–12 km day^−1^ with PoWeR). In contrast, the soleus adapted in a load/volume‐dependent manner, perhaps due to its overall fibre type and recruitment pattern in an exercise model with such a strong endurance training component. Constant‐friction loaded wheel running models, utilizing both low and high resistance, similarly report soleus growth is load‐independent while plantaris mass is unchanged (Leuchtmann et al., 2023; Soffe et al., 2016). Furthermore, the plantaris appears to respond better to wheel running (weighted or unweighted) in rats than mice. This could be due to postural differences during wheel running and rats having slightly larger soleus and plantaris relative to the gastrocnemius than mice, thus contributing more to ankle plantarflexion (Eng et al., 2008; McDonald et al., 1992; Norenberg & Fitts, 2004; Soffe et al., 2016). In other murine exercise models with a strong endurance exercise component, such as forced treadmill running, hindlimb muscle mass and fibre size do not reliably increase, potentially due to higher frequency and total volume of activity with voluntary wheel running compared to treadmill running (Delavar et al., 2014; Kemi et al., 2002; Lee et al., 2018; Okita et al., 2001). Activation of proteins in anabolic cellular signalling cascades could serve as a proxy for muscle growth processes during exercise training. Although phosphorylation status of proteins in the mTOR signalling axis is generally acute by nature, we chose rpS6 as it has been shown to remain phosphorylated up to 24 h after resistance‐exercise stimulus (Langer et al., 2022; Ogasawara et al., 2013). rpS6 is also the immediate downstream target of p70S6K, which is strongly correlated with muscle growth following resistance exercise (Baar & Esser, 1999). Unfortunately, very high variability in our anabolic signalling data limited our ability to observe statistically significant differences in phosphorylation status of rps6 between treatments. Future experiments may choose to explore other anabolic signalling markers, such as p70S6K or direct AMP‐activated protein kinase–mTORC1 interactions to delineate endurance‐ versus resistance‐type signalling events.

Combined resistance and endurance training in humans can also elicit improvements in muscle strength (Schumann et al., 2022). Previously in aged mice, PoWeR‐trained mice displayed greater in vivo peak torque of the GPS complex than SED mice (Dungan et al., 2022). Progressive weighted wheel running also improves peak torque of the GPS in *mdx* mice, a popular model for muscular dystrophy (Call et al., 2010). Similarly, we report peak force of the GPS complex tended to increase in PoWeR and hPoWeR mice. In these investigations, peak force/torque is normalized to the mass of the entire GPS complex, so the less responsive gastrocnemius muscle may be masking any strength adaptations in the much smaller soleus and plantaris muscles. *Ex vivo* measures of soleus isometric tetanic force following unweighted and weighted wheel running in C57BL/10 and *mdx* mice show relatively larger improvements in strength (26–63% vs 10–17%; Call et al., 2010; Hayes & Williams, 1996). Thus, strength measures following wheel running may be better characterized via an in situ or *ex vivo* muscle function test of the soleus and/or plantaris muscles, rather than with the entire GPS complex.

We recognize this current investigation has some limitations. We used male mice as opposed to the female mice traditionally used in previous PoWeR investigations, which may have contributed to lower running distances than commonly observed with PoWeR (Dungan et al., 2019, 2022; Murach, Mobley et al., 2020). We also used solid plastic wheels in our model, whereas PoWeR typically utilizes metal wheels with bars, which may be a preferable running surface. However, previous investigations by our lab report female mice run quite well on the same solid plastic wheels used herein (8–10 km day^−1^ at a similar speed; Zwetsloot et al., 2008). Nevertheless, this investigation further demonstrates the PoWeR training model can be successfully employed in male mice and also with substantially higher loads. Pilot testing should still take place prior to full‐scale studies to characterize running patterns in unique laboratory settings.

To conclude, our novel hPoWeR model may modestly bias progressive weighted wheel running exercise more towards resistance‐type exercise and preserve a ‘faster’ phenotype as shown by a less pronounced fast‐to‐slow fibre type transition due to lower running distances. We observed no remarkable differences in muscle strength adaptation, muscle mass/fibre size, and anabolic signalling between PoWeR and hPoWeR loading protocols. Perhaps extending the wheel running protocol for additional weeks may expose a divergent phenotype between PoWeR and hPoWeR because wheel load and running distance are comparable until the final four weeks. That said, similar training adaptations between the models provides future investigators with flexibility in modifying the PoWeR model loading protocol. Last, although voluntary wheel running still has the advantage of being high‐throughput and low stress for the animal, models such as weighted‐sled pulling should be utilized if an investigator desires to utilize a more purely resistance training model (Zhu et al., 2021).

## AUTHOR CONTRIBUTIONS

The experiments were performed in the Appalachian State University Vivarium and Interdisciplinary Biochemistry Lab. Pieter J. Koopmans performed all experiments, prepared figures and drafted the manuscript. Therin D. Williams‐Frey assisted with daily care of experimental animals and data collection. Pieter J. Koopmans and Therin D. Williams‐Frey conceptualized study design, analysed data, and interpreted results of experiments. Kevin A. Zwetsloot provided supervision and study oversight, edited, and revised manuscript. All authors have read and approved the final version of this manuscript and agree to be accountable for all aspects of the work in ensuring that questions related to the accuracy or integrity of any part of the work are appropriately investigated and resolved. All persons designated as authors qualify for authorship, and all those who qualify for authorship are listed.

## CONFLICT OF INTEREST

The authors have no conflicts of interest to declare.

## Supporting information

Dataset

## Data Availability

All data supporting the results in this paper may be found in the supplemental data file.
